# Circulating HFMD-Associated Coxsackievirus A16 Is Genetically and Phenotypically Distinct from the Prototype CV-A16

**DOI:** 10.1371/journal.pone.0094746

**Published:** 2014-04-15

**Authors:** Wei Wei, Haoran Guo, Jingliang Li, Sangsang Ren, Zhenhong Wei, Wanguo Bao, Xiaoming Hu, Ke Zhao, Wenyan Zhang, Yulai Zhou, Fei Sun, Richard Markham, Xiao-Fang Yu

**Affiliations:** 1 Insititute of Virology and AIDS Research, First Hospital of Jilin University, Changchun, Jilin Province, China; 2 School of life science, Tianjin University, Tianjin, China; 3 College of Pharmacy, Jilin University, Changchun, Jilin Province, China; 4 Department of infectious Diseases, First Hospital of Jilin University, Changchun, Jilin Province, China; 5 Hefei Vocational and Technical College, Hefei, Anhui Province, China; 6 Department of Molecular Microbiology and Immunology, Johns Hopkins Bloomberg School of Public Health, Baltimore, Maryland, United States of America; French-German Advanced Translational Drug Discovery Center, France

## Abstract

Human enteroviruses (HEV) have been linked to hand, foot, and mouth disease (HFMD) in the Pacific and Southeast Asia for decades. Many cases of HFMD have been attributed to coxsackievirus A16 (CV-A16, CA16), based on only partial viral genome determination. Viral phenotypes are also poorly defined. Herein, we have genetically and phenotypically characterized multiple circulating CV-A16 viruses from HFMD patients and determined multiple full-length sequences of these circulating viruses. We discovered that the circulating CV-A16 viruses from HFMD patients are genetically distinct from the proto-type CV-A16 G10. We have also isolated circulating CV-A16 viruses from hospitalized HFMD patients and compared their virological differences. Interestingly, circulating CV-A16 viruses are more pathogenic in a neonatal mouse model than is CV-A16 G10. Thus, we have found circulating recombinant forms of CV-A16 (CRF CV-A16) that are related to, but different from, the prototype CV-A16 G10 that have distinct biological phenotypes.

## Introduction

Hand, foot, and mouth disease (HFMD) is a common infectious disease that mainly affects children ≤5 years of age [1]. HFMD has been endemic in Southeast Asia and the Pacific for decades. Since 2008, a dramatic increase in HFMD prevalence has been reported in mainland China [2]–[8]. Nationwide surveillance reported 488,955 HFMD cases in China in 2008, 1,155,525 cases in 2009, and 1,774,669 cases in 2010 (http://www.moh.gov.cn/publicfiles/business/htm *lfiles/mohjbyfkzj/s3578/201102/50646.htm*). Coxsackievirus A16 (CV-A16) and enterovirus 71 (EV71) have been isolated and identified as the main causes of the large outbreaks of this disease.

Human enteroviruses (HEV) belong to the picornaviridae family, which can be further divided into 12 subgroups on the basis of molecular typing: Enterovirus A, Enterovirus B, Enterovirus C,Enterovirus D, Enterovirus E, Enterovirus F, Enterovirus G, Enterovirus H, Enterovirus J,Rhinovirus A, Rhinovirus B and Rhinovirus C. CV-A16, along with enterovirus 71 (EV71), CV-A2 and CV-A4, are members of the Enterovirus A subgroup. The first and prototype CV-A16 strain, CV-A16 G10, was isolated in South Africa almost 60 years ago [9], [10] and subsequently sequenced in 1994 [11]. CV-A16 viruses contain a single-stranded positive-sense RNA genome of approximately 7.4kb and only a single open reading frame followed by a poly A tract. The CV-A16 RNA genome can be divided into three main regions: a) two non-coding regions, the 5′-UTR and the 3′UTR; b) a structural region, P1 (VP1,VP2,VP3,VP4); and c) the non-structural regions P2 (2A,2B,2C) and P3 (3A,3B,3C,3D) [9]. Infection with CV-A16 occurs mainly through Fecal - oral, respiratory and direct personal contact routes. It can also be spread to newborn infants as a result of intrauterine exposure to the infected pregnant mother [1], [4], [12], [13]. In addition to the classic hand, foot and mouth ulcerations, infection with this virus can also cause myocarditis, intractable shock and poliomyelitis-like paralysis [14], [15].

Based on partial viral sequencing and/or serologic characterization, a significant proportion of the viruses from HFMD patients have been found to be related to the prototype CV-A16 G10 and have thus been classified as CV-A16 strains. Most reports of CV-A16 genetic characterization are based only on the VP1 or VP4 region [3], [16]–[21]. Based on the limited full-length CV-A16 viral sequences available from HFMD patients that were diagnosed after 2008, it has been determined that circulating CV-A16 strains are quite unrelated to the prototype CV-A16 G10 in most parts of the viral genome [22]. To define the full extent of the genetic changes that might exist within the circulating CV-A16 and to study the functional implications of recombination events within nonstructural regions of the genome (especially within viral accessary protein-coding sequences), full-length genome characterization of circulating CV-A16 is urgently needed to facilitate the development of drugs and/or vaccines to prevent and treat this disease.

In the present study, we have sequenced the full-length genome of eight new CV-A16 viruses from HFMD patients. Surprisingly, we have found that prevalent CV-A16 viruses are recombinants with circulating HEV-A viruses in which only one-third of the viral genome is related to CV-A16 G10. Furthermore, significant differences in pathogenicity were observed between the newly identified circulating recombinant CV-A16 viruses and the prototype CV-A16 G10 in a neonatal mouse model.

## Materials and Methods

### Ethics Statement and Sample Selection

This study has been approved by the Ethics Committee of the First Hospital of Jilin University. Written informed consent was obtained from the parents of all the children involved in our study. Eight throat swabs were collected from pediatric patients with clinical diagnoses of CV-A16-related HFMD at the First Hospital of Jilin University in 2010. All specimens were positive for CV-A16 VP1-based characterization assays [9] using a Coxackievirus A16 PCR Kit (DAAN Gene Co., Ltd. of Sun Yat-Sen University).

### Viral RNA Extraction, Reverse Transcription-PCR, and PCR Amplification and Sequencing

The enterovirus sequencing strategy used has been previously described [23]. Extraction of total RNA from throat swabs was performed using TRIzol Reagent (Invitrogen) according to the manufacturer’s instructions. Viral cDNA was generated in a 20-µl reaction volume for 1.5 h at 42°C using Oligo-dT primers and Super-Script II reverse transcriptase (Invitrogen) according to the provided instructions. PCR reactions were performed in 50-µl reaction volumes containing 5 µl of cDNA, 2 U of recombinant Taq DNA polymerase (Takara, Japan), 200 pmol specific forward and reverse primers, 0.4 mM concentrations of deoxynucleoside triphosphates, 20 mM Tris-HCl (pH 8.4), 20 mM KCl, and 1.5 mM MgCl_2_. The cycling conditions consisted of 4 min at 95°C, followed by 35 cycles of 94°C for 30 s to 45 s, 50°C to 55°C for 30 s to 45 s, and 72°C for 1 to 2 min, and finally 72°C for 8 min. The PCR products were examined by agarose gel electrophoresis. The primers used for CV-A16 detection are listed in Table S1. The primers were numbered according to CV-A16 strain SHZH00-1. All amplicons were bidirectionally sequenced. Cycle sequencing was performed with the Big Dye Terminator cycle sequencing kit (Applied Biosystems) and an ABI3730 automated DNA sequencer (Applied Biosystems).

### Sequences from Genbank

The full-length enteroviral sequences were retrieved from Genbank (Genbank number in parentheses): shzh00-1 (AY790926), shzh05-1 (EU262658), GZ08 (FJ198212), coxsackievirus A2 (AY421760), coxsackievirus A3 (AY421761), coxsackievirusA4 (AY421762), coxsackievirus A5 (AY421763), coxsackievirus A6 (AY421764), coxsackievirus A7(AY421765), coxsackievirus A8 (AY421766), coxsackievirus A10 (AY421767), coxsackievirus A12 (AY421768), coxsackievirus A14 (AY421769), coxsackievirus A16 (U05876), enterovirus 71A (U22521), enterovirus 71B (AM396587), enterovirus 71C (DQ341359), enterovirus 76 (AY697458), enterovirus 89 (AY697459), enterovirus 90 (AY697460), enterovirus 91(AY697461), enterovirus 92 (EF667344), enterovirus 68 (AY426531), and poliovirus 1 (V01150).

### Bootscanning and Phylogenetic Analysis

Bootscanning and nucleotide similarity tests were carried out with the Simplot 3.5.1 program [24]. The window and step sizes were determined based on the intent of the analysis and the length of the sequences. The neighbor-joining method and the Kimura 2-parameter model were selected for all bootscanning. To confirm the bootscanning results, genetic algorithm recombination detection (GARD) [25] analysis was also used to detect possible recombination breakpoints within the China CV-A16 sequences. Alignment was achieved with the MEGA4 program [26], using the ClustalW method. The length of nucleotides used for analysis varied, depending on the purpose of the particular analysis, and is clearly indicated in the Results section. All phylogenetic analyses were also performed with the MEGA4 program, using the neighbor-joining method and the Kimura 2-parameter as the model, unless otherwise specified. The phylogeny of each tree was determined for 1,000 replicates with random seeds. To determine potential recombination events, we use both RDP4 [27] and Simplot version 3.5.1 to analyze the recombination. RDP4 was screened with the default settings (standard Bonferroni correction, P values ≤0.05 were considered significant), For the Simplot, a window size of 500 and step size of 20 were used throughout.

### Isolation of Viruses from HFMD Patients and Viral Characterization

Vero cells were used to isolate the Changchun024, Changchun045, and Changchun090 strains of the CV-A16 virus from the throat swabs of patients with HFMD in 2010. The viral samples were diluted in DMEM medium and filter-sterilized using 0.22-µm filters (Millipore, Bedlford, MA); 300 µl of each filtered sample was inoculated into T25 flasks containing approximately 50% confluent Vero cells. Cultures were monitored daily for evidence of cytopathic effect. Culture supernatants from infected Vero cells showing cytopathic effect were collected, aliquoted, viral-titered, and stored at −80°C. Viral titer was determined according to the Reed–Muench method [28]. CV-A16-G10 prototype virus were obtained from the ATCC. Titered viruses were also evaluated for replication in mouse L929 (ATCC #CCL-1), Vero (ATCC, cat. #CCL-81), and human SK-N-SH cells (ATCC, #HTB-11).

### CV-A16-infected Neonatal Mouse Model and Pathological Analysis

One-day-old specific pathogen-free (SPF)-level imprinting control region (ICR) neonatal mice (weighing 1.8–2.0 g, provided by the Experimental Animal Center, College of Basic Medicine, Jilin University) were randomly divided into several groups of three litters per group and 8–10 neonatal mice per litter. The neonatal mice were injected intracerebrally with different viral strains or DMEM medium. Body weight, activity, limb paralysis, morbidity, and death were noted for 21 days post-injection [29], [30]. Protocols involving animals used in this study were approved by the Insititute of Virology and AIDS Research Subcommittee of Research Animal Care.

For immunohistochemical analysis, tissue samples were embedded in optimal cutting temperature (OCT) compound and frozen in liquid nitrogen. The frozen tissue samples were then cut into 4-µm sections, placed on poly-L-lysine-coated glass slides, and fixed in 3.7% paraformaldehyde. The endogenous peroxidase activity of the tissues was inhibited by treatment with hydrogen peroxide (2.5%). CV-A16 antigen was captured by rabbit anti-CV-A16 polyclonal antibody (made in our laboratory) and detected by a Streptavidin-Peroxidase anti-rabbit IgG Kit (Maixin, Fujian), followed by color development with diaminobenzidine for detection of the antigen-antibody reactions.

To detect the viral loads of challenged mice, all tissues (heart, liver, spleen, lung, kidney, brain, intestine, spine skeletal muscle, and hind-limb muscle) were individually weighed and homogenized in sterile phosphate-buffered saline, disrupted by freeze-thawing, and centrifuged. RNA was extracted from the samples using TRIzol (Invitrogen) and viral load was determined by real-time PCR as previously described [31], [32].

### Accession Numbers

The sequences of the eight CV-A16 stains have been entered into the GenBank database under accession numbers KF055238-KF055245.

## Results

### Clinical Features of HFMD Patients Infected with CV-A16 from Changchun, China

Samples from all eight patients with HFMD were collected from the First Clinical Hospital of Jilin University in 2010. Their clinical features are listed in Table 1. All of the patients were children, ranging in age from 2 months to 4 years old. Most of them presented with typical symptoms of HFMD, such as skin rash, herpangina, myoclonic jerks, fever, and vomiting. Some of the patients had complications: one patient (changchun024) had viral meningitis, and another (changchun097) had bronchial pneumonia. Interestingly, one of the eight cases (changchun090) was a 2-month-old child whose mother also had HFMD after delivery.

**Table 1:** Clinical features of eight HFMD patients infected with CV-A16 viruses from Changchun.

	Changchun 024	Changchun 028	Changchun 029	Changchun 045	Changchun 075	Changchun 090	Changchun 097	Changchun 163
**Gender**	Male	Male	Female	Female	Male	Male	Male	Male
**Age**	4Y	2Y	1Y	3Y	1Y	2M	1Y	3Y
**Symptoms**	Skin rash	Skin rash	Skin rash	Skin rash	Skin rash	Skin rash	Skin rash	Skin rash
	Herpangina	Herpangina	Herpangina	Herpangina	Herpangina	Herpangina	Herpangina	Herpangina
	Fever	Fever		Vomit	Myoclonic jerk	Myoclonic jerk	Fever	Fever
	Vomit			Myoclonic jerk				Myoclonic jerk
	Myoclonic jerk							
	Lethargy							
**Complications**	Viral meningitis	No	No	No	No	No	Bronchial pneumonia	No
**Remarks**	No	No	No	No	No	His mother was preinfected	No	No

### Circulating CV-A16 Sequences from Recent HFMD Patients do not Cluster with the Proto-type CV-A16 G10

**Figure 1:**
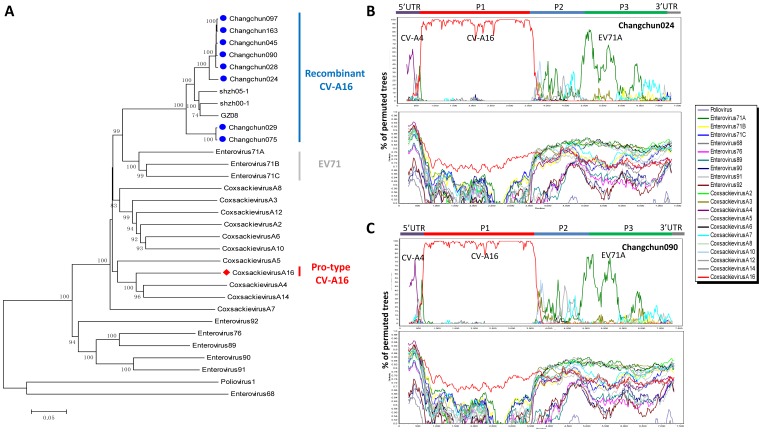
Phylogenetic analysis of eight CV-A16 full-length genomic sequences isolated from HFMD patients in Changchun, China. (A)The complete genomic sequences of 3 other CV-A16 strains from China and all of the 21 HEV reference sequences were retrieved from Genbank. Phylogenetic analysis was conducted using MEGA4 software employing the neighbor-joining method with 1,000 replications and the Kimura 2-parameter model. Bootstrap values greater than 70% are shown. The ▪icon indicates CV-A16 strains isolated from Changchun; the ♦icon indicates the prototype CV-A16-G10. (B) and (C) Identification of recombinant circulating CV-A16 strains of Changchun024 and Changchun029 by bootscanning. (B) Bootscanning analysis of Changchun024 as the query sequence. (C) Bootscanning analysis of Changchun029 as the query sequence. For all HEV-A sequences together with other two outgroups EV68 and poliovirus 1, Changchun024 and Changchun029 showed possible recombination with CA4, CV-A16-G10, and EV71A. Bootscanning was generated with Simplot 3.5.1 software using a sliding window size of 500 bases and step size of 20 bases at a time. The *y* axis shows the percentage of the permuted tree in which the selected HEV virus sequences cluster with the query sequence.

To characterize circulating CV-A16 related to HFMD, full-length viral sequences were obtained from these eight HFMD patients. Phylogenetic analysis indicated that these full-length CV-A16 sequences from Changchun are not closely related to the prototype CV-A16 G10. A neighbor-joining phylogenetic tree was constructed for the eight sequences, along with 19 reference sequences for HEV-A, using EV68 and poliovirus 1 as outliers (Fig. 1A). This analysis confirmed that all the new CV-A16 sequences indeed belonged to HEV-A, which includes CV-A16-G10 (Fig. 1A). However, clustering of these new CV-A16 sequences (Fig. 1A, blue line) was observed with EV71A (BrCr), EV71B (EV71/9/97/SHA89), and EV71C (S10862-SAR-98) (Fig. 1A, grey line), but not with CV-A16 G10 (Fig. 1A, red line). All the circulating CV-A16 (or CV-A16) strains reported since 1998 share the same recombination pattern and form a close cluster in phylogenetic trees (Fig. S1). The only distinct CV-A16 that is available is the prototype G-10 strain.

### Further Analysis of CV-A16 Sequences from China

Since the new HFMD viral sequences contained the CV-A16 VP1 region (data not shown) but clustered within the HEV-A group but not with CV-A16-G10, we examined the viral sequences for evidence of recombination. Bootscanning was performed with the Simplot program to investigate the possibility of recombination within the changchun024 sequence. Various HEV-A sequences were used as reference sequences. The results indicated that the 5′UTR of the changchun024 sequence was closely related to CA4 (Fig. 1B). The P1 region was more closely related to CV-A16-G10. However, the 3′ half of the changchun024 sequence had some similarity to EV71A but not CV-A16-G10 (Fig. 1B). Such patterns were also observed for changchun029 (Fig. 1C) and the other viruses (data not shown).

**Figure 2:**
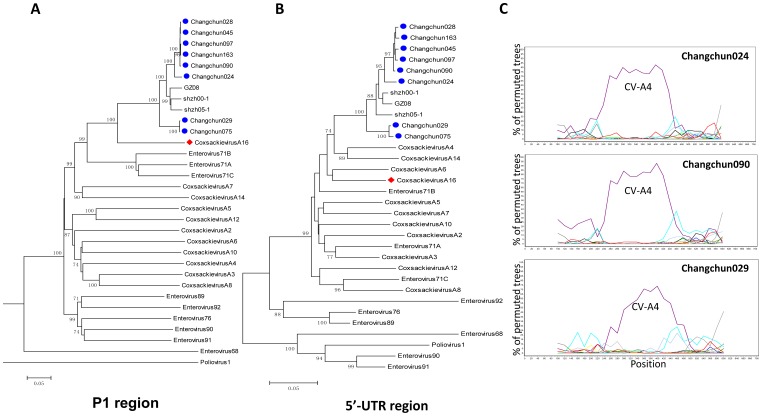
Phylogenetic analysis of the 5′UTR and P1 regions of eight circulating Changchun CV-A16 strains. (A) The neighbor-joining tree was generated based on the 5′UTR sequences (nucleotide 24–718 using CV-A16-G10 sequence as reference) of eight circulating CV-A16 viruses and the HEV reference sequences. (B) The neighbor-joining tree was generated based on the P1 sequences (nucleotides 751–3327 using the CV-A16-G10 sequence as the reference) of eight circulating CV-A16 viruses and reference sequences. The ▪icon indicates CV-A16 strains isolated from Changchun; ♦icon indicates the prototype CV-A16-G10.(C) Detailed bootscanning analysis of the 5′UTR region from CV-A16 strains circulating in Changchun. Bootscanning analysis was performed based on the 5′UTR region sequences (nucleotides 2–718 using the CV-A16-G10 sequence as reference) of Changchun024 and Changchun029, using HEV type A sequences and poliovirus 1 and EV68 as outgroups. A sliding window size of 200 bases and step size of 20 bases was used.

We therefore carefully examined the 5′-UTR. Phylogenetic analysis of the fragment 24–718bp in the 5′UTR showed a small cluster of circulating CV-A16 viruses with CA4 and CA14, with a bootstrap value of 74% (Fig. 2B). On the other hand, the P1 region of circulating CV-A16 viruses clearly clustered with CV-A16 G10 (Fig. 2A). A more detailed bootscanning of the 5′-UTR of changchun024 and changchun029 with a smaller window of 200bp showed a dominant CA4 sequence, but not CA14, in the 5′-UTR of changchun024 and changchun029 viruses (Fig. 2C). Similar results were observed for the other circulating Changchun recombinant CV-A16 viruses (data not shown).

### Detailed Analysis of the P2 and P3 Regions

**Figure 3:**
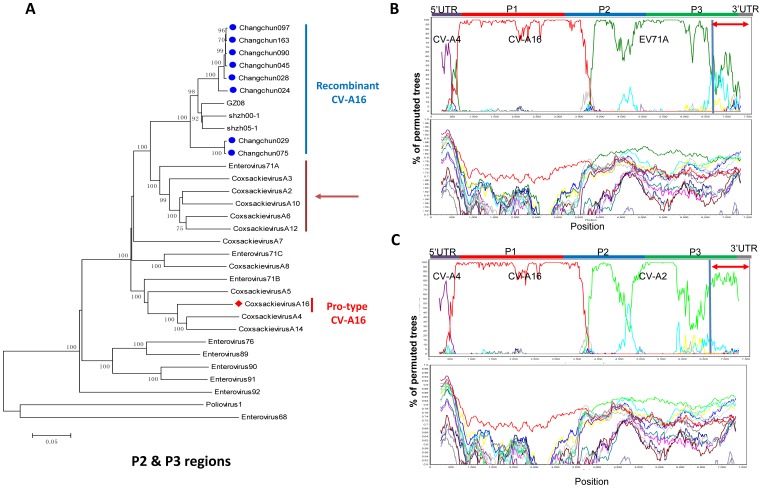
Phylogenetic analysis of P2 and P3 regions of eight circulating Changchun CV-A16 strains. (A) Phylogenetic analysis of the P2 and P3 sequences of circulating CV-A16 stains (nucleotides 3341–7328 using the CV-A16-G10 sequence as the reference). Only strong bootstrap values (>70%) are shown.▪icon indicates CV-A16 strains isolated from Changchun; ♦icon indicate the prototype CV-A16-G10. (B) Bootscanning analysis of Changchun024 complete genomic sequences was performed with all HEVA sequences except CV-A2, CV-A3, CV-A6, CV-A10 and CV-A12. (C) Bootscanning analysis of Changchun024 was performed with all HEVA sequences except EV71,CV-A3, CV-A6, CV-A10 and CV-A12. (D) Bootscanning analysis of Changchun024 was performed with all HEVA sequences except EV71, CV-A2, CV-A3, CV-A6 and CV-A10. Poliovirus 1 and enterovirus 68 were used as outgroups. A window size of 500 bp and step size of 20 bp at a time were used.

The bootscanning results indicated that the P2 and P3 regions of circulating CV-A16 viruses showed no significant similarity to CV-A16 G10 (Fig. 1B,1C). To help pinpoint the possible origin of the P2 and P3 regions of the circulating CV-A16 viruses, the 3′ sequences were further examined. A more detailed phylogenetic analysis of the P2 and P3 (Fig. 3A) regions demonstrated clustering of circulating CV-A16 viruses with a group of HEV-A viruses, including EV71A, CV-A2, CV-A3, CV-A6, CV-A10, and CV-A12, but not CV-A16 G-10. Bootscanning analysis further confirmed the possible relationship of the P2 and P3 regions of changchun024 to EV71A (Fig. 3B), CV-A2 (Fig. 3C), CV-A12 (Fig. 3D) and CV-A3, CV-A6, CV-A10 (Fig. S2). These results indicated a possible recombination in the P2 and P3 regions between CV-A16 and a virus that is related to EV71A, CV-A2, CV-A3, CV-A6, CV-A10, or CV-A12. Similar results were also observed for other circulating Changchun recombinant CV-A16 viruses (data not shown).

In order to reconfirm our conclusion, we used a series of algorithms in the RDP program, including RDP,GENECONV, BootScan, MaxChi, Chimaera, SiScan, PhylPro, LARD, and 3Seq, to detect the recombination events. The results confirmed those obtained with Bootscan in the Simplot program: when the sequence of changchun024 was analyzed in the absence of reference sequences CV-A2, CV-A3, CV-A6, CV-A10, and CV-A12, seven algorithms detected significant recombination with EV71A at the P2-P3 region; when the sequence of changchun024 was analyzed in the absence of reference sequences EV71A, CV-A3, CV-A6, CV-A10, and CV-A12, recombination was detected in two regions: five algorithms detected recombination with CV-A2 in the P2 region and four algorithms detected recombination with CV-A2 in the P3 region. This was consistent with the bootscan results. Of particular interest was the recombination with CV-A2 detected in the 3D region, which has not been previously reported. In a similar fashion, when the sequence of changchun024 was analyzed in the absence of reference sequences EV71A, CV-A2, CV-A3, CV-A6 and CV-A10, recombination with CV-A12 was detected using six algorithms. All of the recombination events had high P-values (Table 2).

**Table 2:** Summary of Changchun024 recombination events detected by RDP4.

Reference sequences	Break points	P-value
Poliovirus,EV68, EV71B,EV71C,EV76,EV89,EV90,EV91,EV92,CV-A4,CV-A5,CV-A7,CV-A8,CV-A14,CV-A16, (**EV71A**)	3751–5856	RDP(2.431×10^−07^); GENECONV(1.972×10^−01^);Bootscan(4.046×10^−09^); Maxchi(6.648×10^−12^);Chimaera(3.961×10^−10^); SiSscan(1.886×10^−19^);3Seq(6.068×10^−04^)
Poliovirus,EV68, EV71B,EV71C,EV76,EV89,EV90,EV91,EV92,CV-A4,CV-A5,CV-A7,CV-A8,CV-A14,CV-A16, (**CV-A2**)	3661–5849	RDP(2.257×10^−05^); Bootscan(1.295×10^−08^);Maxchi(5.429×10^−11^); Chimaera(1.300×10^−08^);SiSscan(2.254×10^−19^)
	6571–7330	RDP(3.045×10^−02^); Bootscan(5.457×10^−03^);Chimaera(6.725×10^−01^); SiSscan(5.353×10^−09^)
Poliovirus,EV68, EV71B,EV71C,EV76,EV89,EV90,EV91,EV92,CV-A4,CV-A5,CV-A7,CV-A8,CV-A14,CV-A16 (**CV-A12**)	4002–5792	RDP(3.641×10^−05^); Bootscan(6.119×10^−08^);Maxchi(2.271×10^−08^); Chimaera(1.082×10^−09^);SiSscan(1.295×10^−16^); 3Seq(3.954×10^−01^)

### Biological Characterization of CRF CV-A16 from HFMD Patients

**Figure 4:**
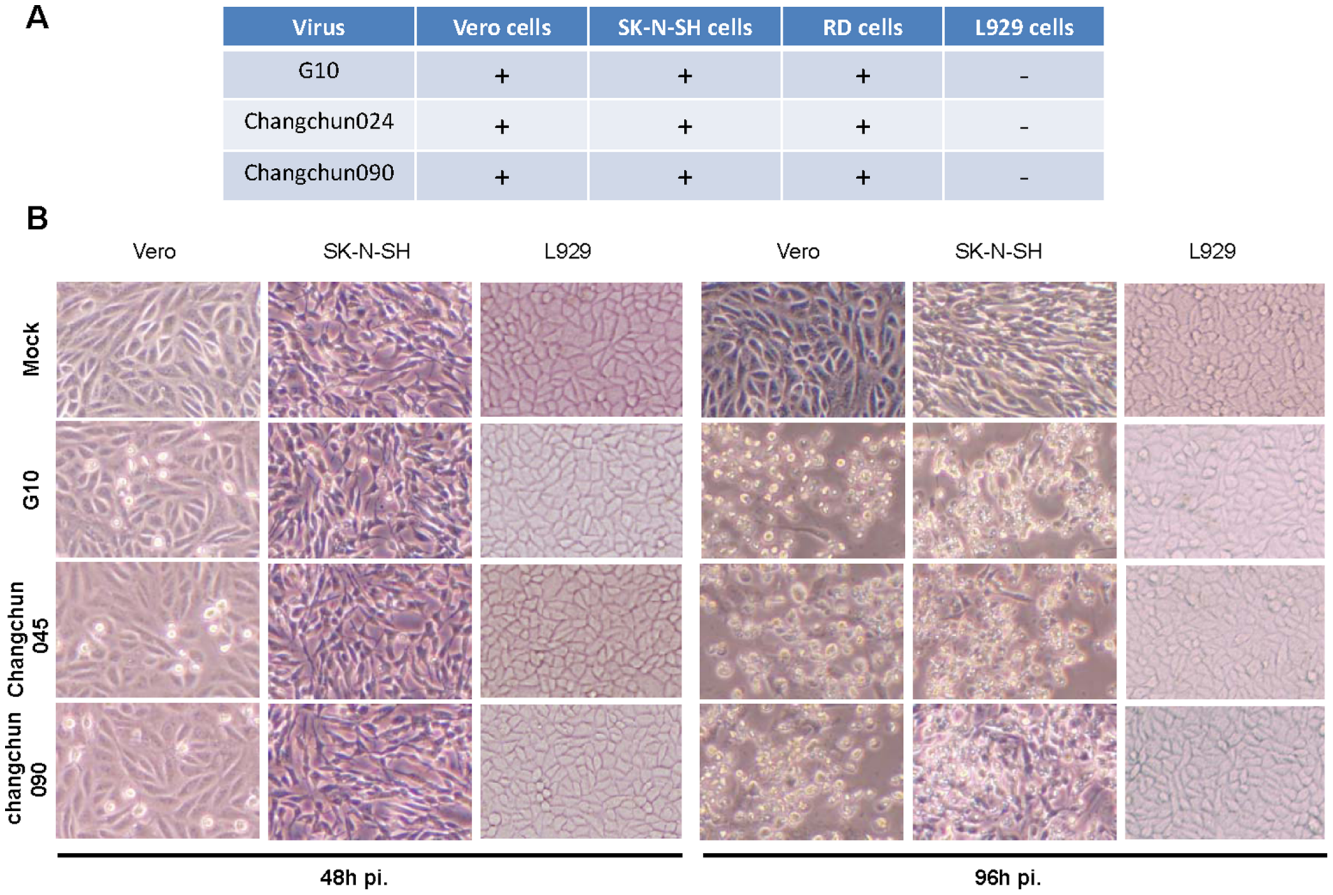
Tissue culture replication of CV-A16 viruses. Viral replication was monitored by the induction of CPE by the prototype CV-A16-G10 and the circulating Changchun CV-A16 strains. Viruses were isolated from infected Vero cells. Viral titers were determined as previously described (Ref). Equal amounts of virus were used to infect Vero, SK-N-SH, RD and L929 cells. Viral replication resulting in CPE of >90% of cells was recorded as+(A). (B) Representative CPE caused by the prototype CV-A16-G10 and Changchun circulating CV-A16 viruses are shown. Images were obtained using phase contrast microscopy (Olympus IX51, Center Valley, PA).

Since circulating recombinant CV-A16 viruses differ from CV-A16 G10 genetically, we were interested in whether these viruses also differed in their biological properties. We evaluated viral replication in multiple cell lines. Like CV-A16 G10, CV-A16 changchun045 and CV-A16 changchun090 replicated and caused a cytopathic effect in African green monkey kidney Vero cells, a cell line sensitive to most enterovirus infections (Fig. 4A, 4B). The CPE started at 48 h after infection and peaked at 96 h after infection (Fig. 4B). A previous report has indicated that CV-A16 G10 cannot infect the mouse fibroblast cell line L929 [33]. Our data indicated that CV-A16 changchun045 and CV-A16 changchun090 also did not replicate or cause a cytopathic effect in L929 cells (Fig. 4B). When similar viral titers of various CV-A16 viruses were used to infect human neuroblastoma SK-N-SH cells, we found that that all the Changchun-circulating CV-A16 strains and CV-A16 G10 could induce an obvious cytopathic effect by 96 h post-infection (Fig. 4B). Thus, our results indicated that all the Changchun recombinant CV-A16 and CV-A16-G10 strains were unable to infect L929 cells, but they had the same ability to infect and induce cell death in human neuroblastoma cells.

### Different Pathogenetic Outcomes in Neonatal Mice for the Prototype CV-A16-G10 and Circulating Recombinant forms of CV-A16

**Figure 5:**
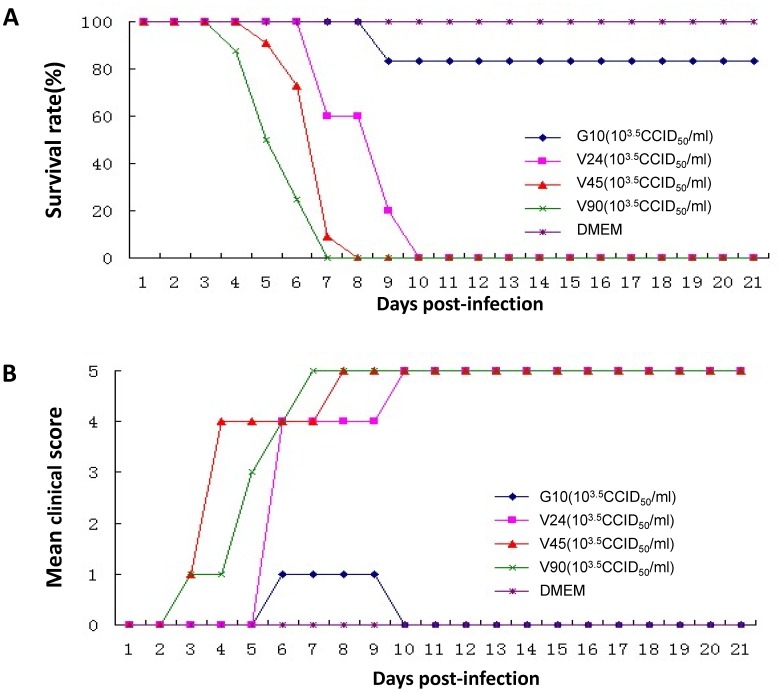
Disease and mortality rate differences in newborn mice caused by different strains of CV-A16. Differences in virulence between the prototype CV-A16-G10 and circulating recombinant forms of CV-A16 in neonatal mice were compared. One-day-old ICR mice were intracerebrally inoculated with 10^3.5^ TCID50/ml G10, Changchun024, Changchun045, Changchun090 viruses in DMEM medium. The negative control mice were given medium instead of the virus suspension. The survival rates (A) and clinical scores (B) were monitored and recorded daily after infection for 21 days. The clinical score was graded as follows: 0: no abnormalities; 1: lethargy and inactivity; 2: wasting; 3: limb shake weakness; 4: hind-limb paralysis; 5: moribund or dead. Each group contained six to ten mice.

**Figure 6:**
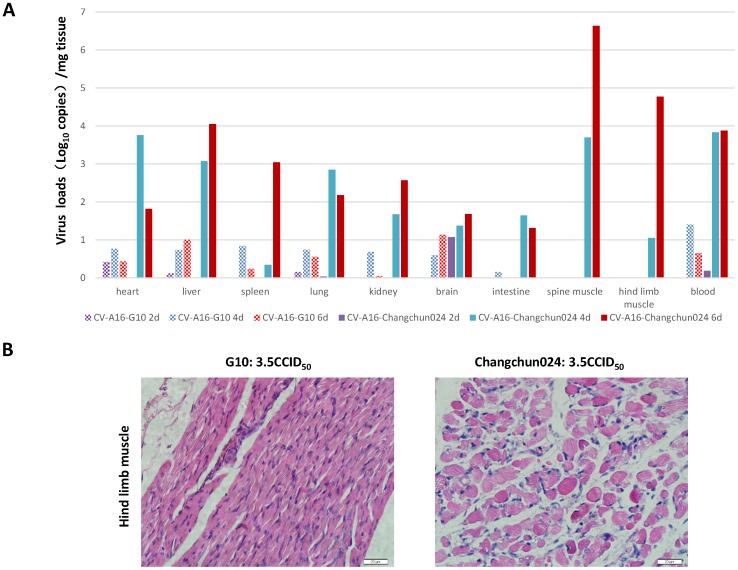
Measures of tissue and blood viral loads of CV-A16-infected newborn mice. (A) One-day-old mice were intracerebrally inoculated with Changchun 024 or prototype G10 viruses. Virus loads were assessed by real time quantitative PCR in samples of the heart, liver, spleen, lung, kidney, brain, intestine, spine muscle, hind limb muscle and blood from the infected newborn mice. Samples were collected at the indicated time; (B) Immunohistochemical analysis of hind limb muscle from Changchun024 or CV-A16-G10 challenged newborn mice.

As described above, the Changchun circulating CV-A16 strains are recombinant and differ from the prototype CV-A16 G10 in most regions of the viral genome. Although viral replication in certain cell lines appeared to be unaltered by the recombination, whether these recombinants have different pathogenetic potential still needed to be evaluated. To address this issue, we established a mouse model of lethal CV-A16 infection in order to compare the pathogenesis of the prototype CV-A16 G10 and the Changchun-circulating CV-A16 strains. We found that CV-A16 changchun024, which was isolated from a HFMD patient with viral meningitis, was highly pathogenic in newborn mice. To evaluate any possible differences in viral pathogenesis, we established a grading system for evaluating the clinical condition of the mice: no abnormalities (grade 0), lethargy and inactivity (grade 1), wasting (grade 2), limb shaking and weakness (grade 3), hind-limb paralysis (grade 4), and moribund or dead (grade 5) [29]. The negative control, DMEM medium, and CV-A16 changchun024 in DMEM medium were injected intracerebrally into one-day-old neonatal mice. The negative control group showed no disease symptoms and had a 100% survival rate. The mice that were infected with CV-A16 changchun024 became sick on day 5 post-infection, with a mean clinical score of grade 4, and all were dead by day 10 (Fig. 5B). Similarly, all mice that were challenged with changchun045 and changchun090 developed clinical symptoms with a clinical score up to 4, and all were dead by day 8 (Fig. 5B). In contrast, the clinical symptoms of G10-infected mice were much less severe than those of mice infected with the circulating CV-A16 viruses (Fig. 5B). The majority of the mice infected with CV-A16 G10 survived after infection up to day 21(Fig. 5A). Our results indicated that in this lethal-challenge mouse model, recombinant circulating CV-A16 viruses were more pathogenic than the prototype CV-A16 G10. Consistent with these findings, the viral loads of different organs and blood from the CV-A16-infected newborn mice showed that dramatically higher viral loads were present in Changchun024-infected newborn mice compared to the organs of mice infected with the prototype CV-A16 G10 viruses (Fig. 6A). In particular, our data clearly indicated that circulating recombinant form CV-A16 had significantly higher replication activity in the spine muscle and hind limb muscle. Further immunohistochemical analysis also supported our results in the hind limb muscle of challenged mice (Fig. 6B). In general, CV-A16 Changchun024 had higher virus loads than CV-A16-G10 in all tested samples of organs and blood. In particular, the CV-A16 Changchun024 loads in the spine muscle and hind limb muscle from infected mice were much higher (>10^5^ copies/mg tissue) than the loads in these muscles from CV-A16 G10-challenged mice, which contained no significant detection of CV-A16 G10. These findings are consistent with Changchun024 being much more virulent in this model system.

## Discussion

Coxsackievirus A16, like other enteroviruses, shows a high mutation rate during viral replication due to the deficiency of proofreading activity. In the current study, we have determined eight full-length sequences of CV-A16 viruses from recent HFMD patients. Our study revealed significant differences in the genomic structures between the prototype CV-A16 G10 and the currently circulating CV-A16 viruses. Further analysis of several CRF CV-A16 viruses isolated from hospitalized patients revealed similar viral phenotypes, which were distinct from that of CV-A16 G10.

We first demonstrated that circulating CV-A16 viruses from recent HFMD patients in China are actually complex recombinant viruses involving multiple type A HEV, including CA4, CV-A16, and possibly EV71, CV-A2, CV-A3, CV-A6,CV-A10 or CV-A12. The 5′UTR region of these viruses had the highest similarity to CV-A4. Most of the structural protein (P1) region resembled that of the prototype CV-A16 G10 strain. However, the non-structural protein domains (P2 and P3) showed similarity to multiple type A HEV viruses but not CV-A16 G10.

The detailed characterization of eight new CV-A16-related full-length viral sequences as well as three earlier CV-A16-related sequences from China confirm the previous report [22] that the 5′UTR region of the viral genome has the highest similarity to CA4. Interestingly, CA4 has been detected in HFMD patients in Zhejiang province (central China) [22] and in Gansu province (northwest China) [7]. Although the P1 regions of the currently circulating recombinant CV-A16 viruses are mostly related to the prototype CV-A16-G10, they still differ from CV-A16 G10 by almost 20% at the amino acid level. On the other hand, the P1 regions of all the circulating CV-A16 viruses have divergences from each other of less than 10%.

The non-structural protein domains (P2 and P3) of circulating CV-A16 viruses are most divergent from the same regions in CV-A16 G10. The origin of these P2 and P3 regions is also not clear. Recent reports have suggested that circulating CV-A16 strains are recombinant viruses that have recombined with different HEV-A type viruses. However, these reports are not in total agreement concerning the possible origin of the P2 and P3 regions in these circulating recombinant CV-A16 viruses. Chan Yoke-Fun *et al.* and Cyril C.Y.Yip *et al*. claimed that the P2 and P3 regions of circulating CV-A16 strains in China were mainly from EV71A [34], [35]. On the other hand, Ke Zhao *et al*. found that only a 1.5-kb fragment (4,406–5,585 bp) of these nonstructural domains was similar to EV71A [34], [35]. In the present study, we have observed a similar pattern for the newly identified circulating recombinant CV-A16 viruses (Fig. 2). Based on phylogenetic analysis, the P2 and P3 regions of the circulating recombinant CV-A16 viruses are actually more closely related to a group of HEV type A viruses, including EV71A, CV-A2, CV-A3, CV-A6, CV-A10, and CV-A12 (Fig. 2A). In this case, bootscanning results can be significantly influenced by which reference viral sequence is used (Fig. 2B; Fig. S1). These findings emphasize the importance of using a more complete set of HEV reference sequences for bootscanning analysis. Since the exact functions of the nonstructural region (P2 and P3) in viral replication and pathogenesis are still not well defined, future studies should be encouraged to explore how and why so many enteroviruses share a similar nonstructural region.

Despite the fact that only a third of the viral genome of the circulating CV-A16 viruses shares similarity with CV-A16 G10, these viruses had a similar replication capacity in multiple mammalian cell lines. All the viruses replicated well in human neuroblastoma SK-N-SH cells and African green monkey kidney Vero cells, and none could efficiently replicate in the mouse fibroblast cell line L929. Thus far, two cellular receptors have been identified for CV-A16 and EV71 viruses: human scavenger receptor B2 (SCARB2) and P-selectin glycoprotein ligand-1(PSGL-1) [33], [36], [37]. Unlike PSGL-1, which is mainly expressed in lymphoid cells, SCARB2 is widely expressed in both EV71 and CV-A16 target cells. However, mouse SCARB2 (unlike human SCARB2) could not support efficient EV71 or CV-A16 infection in L929 cells. It is likely that the cellular tropism of the circulating CV-A16 viruses is similar to that of the prototype CV-A16 G10.

Surprisingly, circulating CV-A16 viruses were more pathogenic than the prototype CV-A16 G10 in neonatal mice despite similar replication patterns in tissue culture. Circulating CV-A16 viruses induced more disease-related symptoms in neonatal mice than did CV-A16 G10. Furthermore, whereas circulating CV-A16 viruses produced a 100% death rate in infected mice, the majority of the CV-A16-G10-infected mice survived. To our knowledge, this is the first example of a positive or negative effect of a CV-A16 recombinant on viral pathogenesis in an animal model. It is well known that many enterovirus recombinants have gained activity related to viral replication or spread in animal models, and, while the implications of these findings for human disease have yet to be determined, our results raise the possibility of evolution to increased virulence. The 5′UTR and the nonstructural P2/P3 regions showed the most difference between CV-A16 G10 and the circulating recombinant CV-A16 viruses. The contribution of these regions to viral pathogenesis should be explored in future experiments. In addition, it is still not clear if the significantly higher viral loads in the spine muscle and hind limb muscle of CRF CV-A16-infected newborn mice are due to specific receptor expression or more suitable viral replication environments; similar results were also detected in our CV-A16 vaccine candidate studies [32]. It is equally interesting to note that multiple CV-A16 virus strains could cause a lethal infection in the neonatal mice, despite the fact that mouse PSGL-1 and SCARB2 are not expected to mediate efficient replication of CV-A16 viruses in these mice. Our findings raises the question of whether additional cellular receptors contribute to CV-A16 infection in our neonatal mouse model.

To date, there is still no effective vaccine or drug against CV-A16 infection. The significant differences among CV-A16 viruses in cellular infection systems and animal models may be important factors for vaccine development and evaluation. The establishment of animal models of lethal CV-A16 infection that could distinguish between different CV-A16 viral strains would also be critical for subsequent studies of the pathogenesis of CV-A16. Further elucidation of the mechanisms that govern the differences in these different model systems may also shed light on our understanding of CV-A16 enterovirus recombination and viral pathogenesis.

## Supporting Information

Figure S1 Phylogenetic analysis of full-length genomic sequences of CV-A16 viruses.
(TIFF)

Figure S2 Characterization of circulating Changchun CV-A16 viruses with a similar origin as other enteroviruses in the P2 and P3 regions. (A) Bootscanning analysis of Changchun024 was performed with all HEVA sequences except EV71,CV-A2,CV-A6,CV-A10 and CV-A12. (B) Bootscanning analysis of Changchun024 was performed with all HEVA sequences except EV71,CV-A2,CV-A3,CV-A10 and CV-A12. (C) Bootscanning analysis of Changchun024 was performed with all HEVA sequences except EV71,CV-A2,CV-A3,CV-A6 and CV-A12.
(TIFF)

Table S1 A list of primers used for CA16 sequence PCR amplifications.
(DOC)
