# Supplementary material for: Circulating HFMD-Associated Coxsackievirus A16 Is Genetically and Phenotypically Distinct from the Prototype CV-A16
Source: PLoS One. 2014 Apr 15;9(4):e94746. doi: 10.1371/journal.pone.0094746 (PMC3988102; doi:10.1371/journal.pone.0094746)
Supplement: Table S1 — A list of primers used for CA16 sequence PCR amplifications. (DOC) [file pone.0094746.s003.doc]

Table S1  A list of primers used for CA16 sequence PCR amplifications.
Primers	Sequences (5'- to 3'- terminus)	
CVA16-1F	TTAAAACAGCCTGTGGGTTGTTC	
CVA16-1R	CATCTGTGTCTGGGCAGTA	
CVA16-2F	ATTTACTGACCCTGTCATG	
CVA16-2R	CCATCATCAGTAGTAAGAAAC	
CVA16-3F	CACCATAGCTCCCATGTG	
CVA16-3R	TTGGCAGCTGTAGGTAGTAC	
CVA16-4F	TACATCGTGGCTTTGGC	
CVA16-4R	CAAGGTGCCGATTCACTA	
CVA16-5F	GTTATCCCACCTTCGGAGAGCA	
CVA16-5R	GAGCTAAATATGACACATTACCAAACA	
CVA16-6F	CTCACAGCTACGCTTGC	
CVA16-6R	GCTAGGCGATCTCGCA	
CVA16-7F	GTTGTGTCTCTTGTCTATGTC	
CVA16-7R	TTGCTCAAAATCAACCTC	
CVA16-8F	TACAATTTCCCCACAAAG	
CVA16-8R	TGCTATTCTGGTTATAACAA	
